# Is Creutzfeldt-Jakob disease transmitted in blood?

**DOI:** 10.3201/eid0302.970208

**Published:** 1997

**Authors:** M N Ricketts, N R Cashman, E E Stratton, S ElSaadany

**Affiliations:** Bureau of Infectious Diseases, Laboratory Centre for Disease Control, Ottawa, Ontario, Canada. mricketts@hpb.hwc.ca

## Abstract

Creutzfeldt-Jakob disease (CJD) has been considered infectious since the mid-1960s, but its transmissibility through the transfusion of blood or blood products is controversial. The causative agent's novel undefined nature and resistance to standard decontamination, the absence of a screening test, and the recognition that even rare cases of transmission may be unacceptable have led to the revision of policies and procedures worldwide affecting all facets of blood product manufacturing from blood collection to transfusion. We reviewed current evidence that CJD is transmitted through blood.


### Is Creutzfeldt-Jakob Disease Transmitted in Blood?

**Maura N. Ricketts,* Neil R. Cashman,† Elizabeth E. Stratton,* Susie ElSaadany***
*Laboratory Centre for Disease Control, Health Canada, Ottawa, Ontario, Canada; and †Montreal Neurological Institute, Montreal, Canada

Creutzfeldt-Jakob disease (CJD) has been considered infectious since the mid-1960s, but its transmissibility through the transfusion of blood or blood products is controversial. The causative agent's novel undefined nature and resistance to standard decontamination, the absence of a screening test, and the recognition that even rare cases of transmission may be unacceptable have led to the revision of policies and procedures worldwide affecting all facets of blood product manufacturing from blood collection to transfusion. We reviewed current evidence that CJD is transmitted through blood.

Creutzfeldt-Jakob disease (CJD), a rare neurodegenerative disorder, affects 0.5 to 1 persons per million population worldwide each year (1-8). CJD is a human spongiform encephalopathy; others are kuru, which is associated with ritualistic cannibalism in the Fore tribe of Papua New Guinea; Gerstmann-Sträussler-Scheinker syndrome, an inherited disorder; and fatal familial insomnia, inherited as an autosomal-dominant trait. Animal spongiform encephalopathies include scrapie, bovine spongiform encephalopathy (BSE), transmissible mink encephalopathy, and wasting disease of elk most frequently referred to as transmissible spongiform encephalopathies; other names such as prion dementias, transmissible degenerative encephalopathies, and infectious cerebral amyloidoses are also used.

The classic clinical symptoms of CJD are rapidly progressive presenile dementia, myoclonus, and progressive motor dysfunction. No treatment is available, and survival averages less than 1 year (most often 2 to 6 months) (9). Diagnosis is based on symptoms, electroencephalograms, and neuropathologic tests (10,11).

The pathophysiology of CJD is incompletely understood, although it is known that in persons with the disease, the normal soluble prion protein ($PrP^C$) is conformationally shifted into a more stable, less soluble ß-pleated protein. The term $PrP^{CJD}$ indicates the abnormal isoform found, with a variety of distribution patterns in the brain of persons with CJD. Limited proteolysis of $PrP^{CJD}$ by Western blot analysis shows four patterns of protease-resistant prion protein (12,13).

No screening assay is available to detect PrP in asymptomatic persons. Cerebrospinal fluid protein markers can distinguish CJD from other neurodegenerative disorders in certain settings (14,15). However, they are not CJD-specific and are not markers of PrP. The etiologic agent is believed to be a prion (proteinaceous infectious particle), although viral etiologies have been proposed (16-18). Sporadic CJD may also result from the spontaneous conversion of $PrP^C$ into $PrP^{CJD}$ or from somatic mutation in the prion protein gene.

Three epidemiologic forms of CJD are well recognized: sporadic (the most common), familial, and infectious/iatrogenic. The familial form (5% to 10% of cases) results from mutations in the coding sequence of the PrP gene located on chromosome 20. Polymorphisms at codon 129 have been correlated with genetic susceptibility to human prion diseases (19). Fewer than 1% of human cases of CJD are iatrogenic (20). A new variant (nv-CJD), which occurred temporally in association with BSE in cattle in the United Kingdom, was recently reported (21); a direct association with food consumption remains

Address for correspondence: Maura N. Ricketts, Blood-borne Pathogens Division, Bureau of Infectious Diseases, Laboratory Centre for Disease Control, LCDC Building, PL 060 3E1, Ottawa, Ontario, K1A 0L2 Canada; fax: 613-954-6668; e-mail: mricketts@hpb.hwc.ca.

uncertain. Possible transmission of CJD through receipt of blood or blood products is a concern. Routes of iatrogenic transmission are summarized below, followed by a discussion of possible bloodborne transmission.

### Iatrogenic CJD

Although first described in the 1920s, CJD was not considered a transmissible disease until 1966 when kuru was shown to be transmissible (22). In 1968, CJD was confirmed to be a transmissible spongiform encephalopathy when it was shown to be transmitted to chimpanzees (23). Virtually every case of CJD attributed to infection is iatrogenic; transmission between humans has been clearly demonstrated during neurosurgical procedures with contaminated instruments and through central nervous system tissue and extract transfer. Worldwide, more than 100 cases of transmissible CJD have been detected, and new cases continue to appear (Table 1; 27).

The first report of suspected iatrogenic CJD was published in 1974 (24). Animal experiments showed that corneas of infected animals could transmit CJD, and the causative agent spreads along visual pathways (25,26). A second case of CJD associated with a corneal transplant was reported without details (27). In 1977, CJD transmission caused by silver electrodes previously used in the brain of a person with CJD was first reported (28). Transmission occurred despite decontamination of the electrodes (later shown to transmit disease to experimental primates [29]) with ethanol and formaldehyde. Retrospective studies identified four other cases likely of similar cause (30-32). The rate of transmission from a single contaminated instrument is unknown, although it is not 100% (31). In some cases the exposure occurred weeks after the instruments were used on a person with CJD.

CJD was first reported in a recipient of a dura mater transplant in 1987 (33); a second case was identified in 1989 in a 25-year-old man from New Zealand, who also received dura mater (34). Because the same company produced dura mater for both patients, the dura mater was suspected as the source of iatrogenic CJD. Approximately 15 case reports have been published, providing information on 20 cases associated with dura mater transplant; a recent review article indicates that 25 such cases have occurred throughout the world (Australia, Canada, Germany, Italy, Japan, New Zealand, Spain, the United Kingdom, and the United States) (27). However, Japan has very recently reported more than 40 cases of CJD in dura mater recipients (J. Tateishi, Pers. Comm.). Most dura mater cases have been associated with a single manufacturer whose manufacturing processes were inadequate to inactivate the prion agent. This, combined with pooling of the dura mater has led to the relatively large number of cases. The earliest reported transmission occurred in a patient who received a dura mater transplant in 1969 (35). Although recommendations to reduce the risk were made in 1987, cases have continued to appear because of the long incubation period. Notably, one of the most important epidemiologic characteristics of dura mater transmissions is the young age of the first reported cases.

By 1985, a series of case reports in the United States showed that when injected, cadaver-extracted pituitary human growth hormone could transmit CJD to humans (36). Shortly thereafter, it was recognized that human gonadotropin administered by injection could also transmit CJD from person to person (37).

Strain typing based on molecular PrP analysis and a polymorphism (methionine/methionine, valine/valine or methionine/valine) found at codon 129 of the PrP gene has been proposed as a tool for distinguishing between iatrogenic and sporadic CJD (12,13). Homozygosity at this site may predispose a person to acquired forms of

Table 1. Transmissible cases of Creutzfeldt-Jakob Disease

| Mode of infection | No. of patients | Agent entry into brain | Mean incubation period (range) |
|---|---|---|---|
| INSTRUMENTATION | | | |
| Neurosurgery | 4 | Intracerebral | 20 months (15-28) |
| Stereotactic EEG | 2 | Intracerebral | 18 months (16-20) |
| TISSUE TRANSFER | | | |
| Corneal transplant | 2 | Optic nerve | 17 months (16,18) |
| Dura mater implant | 25 | Cerebral surface | 5.5 years (1.5-12)) |
| TISSUE EXTRACT TRANSFER | | | |
| Growth hormone | 76 | Hematogenous | 12 years (5-30) |
| Gonadotrophin | 4 | Hematogenous | 13 years (12-16) |

EEG = electroencephalogram
Table taken from (27)

CJD, may lead to shorter incubation periods, and has been associated with various phenotypes of the disease (11,38). The proportion of polymorphism among persons with sporadic CJD is more similar to the proportion of polymorphism among persons with iatrogenic CJD than to that in the general population (Table 2), which suggests that simple stochastic events do not fully explain sporadic CJD; were that so, the distribution of homozygosity would be the same in both healthy controls and persons with sporadic cases. The clinical symptoms in patients with iatrogenic and with sporadic CJD have been compared and are indistinguishable (18).

Table 2. Amino acid phenotypes for codon 129 in patients with iatrogenic Creutzfeldt-JakobDisease

| Tested groups | Met/Met (%) | Met/Val (%) | Val/Val (%) | Homozygous (%) | Total No. tested (n) |
|---|---|---|---|---|---|
| Sporadic CJD (38) | 78 | 12 | 10 | 88 | 73 |
| All Iatrogenic | 60 | 11 | 29 | 89 | 63 |
| Cases (27) | | | | | |
| CNS route of infection | 80 | 10 | 10 | 98 | 20 |
| Peripheral route of infection | 51 | 12 | 37 | 88 | 43 |
| Healthy controls (27) | 37 | 51 | 11 | 49 | 261 |
| Healthy controls (38) | 48 | 42 | 10 | 58 | 1397 |

Met = methionine; Val = valine

### Is CJD Transmitted by Blood Transfusions?

### Animal Experimentation Data

Human CJD has been reported to be transmitted to mice by injecting blood from human patients directly into mouse brain (39,40). However, this evidence has not been widely duplicated by other laboratories, and a comprehensive review of three decades of research with nonhuman primates by the National Institutes of Health indicated that the blood of humans with CJD injected either peripherally or centrally into primates did not transmit CJD (41).

Some evidence indicates that blood of experimentally infected animals contains an infective agent. PrP infectivity resides predominantly or exclusively in lymphocytes and monocytes rather than in granulocytes (42,43); there has been no evidence of infectivity in erythrocytes, platelets, or plasma, but low infectivity has not been completely excluded. Animal studies have demonstrated that the agent causing scrapie replicates first in the spleen and other lymphoid tissues but reaches highest titer in the brain, where it results in the clinical appearance of disease (44). Hence, peripheral tissues in contact with blood also harbor PrP infectivity. Animal transmission data indicate that human spleen, lymph nodes, serum, and cord blood are irregularly infective for animals, although few cord blood samples have been tested (27). Brown has recently reported transmission of mouse scrapie using fractionated blood administered by intracerebral inoculation (P. Brown, Pers. Comm.). Studies of experimental CJD in guinea pigs and mice have shown that the infectious agent is present in the brain, viscera, and blood before clinical disease develops (43,45).

Several factors must be considered in reviewing animal evidence regarding transmission of CJD in blood: the level of PrP infectivity of the study tissue, the species barrier, and the route of administration. Human CJD can be readily transmitted to nonhuman primates (low species barrier) by intracranial injection (high efficiency of the infective route) of contaminated brain (high titer of infectivity). Human CJD is not readily transmitted to dissimilar species (such as rodents) and is even less readily transmitted when low-titer tissues (e.g., blood cells) and/or a low efficiency route of inoculation is used. In animals, peripheral inoculation of brain tissue transmitted CJD only irregularly, but the incubation periods were comparable to central administration (41). Barriers to transmission also include the difficulties in infecting peripheral cells and crossing the blood brain barrier and possibly the lack of structural similarity between peripheral $PrP^{CJD}$ and brain $PrP^{C}$. Strain variation may also contribute to the difficulties of transmission to animals. Deslys et al. suggest that the route of inoculation may induce strain differentiation, which then facilitates subsequent transmission by the same route (46). Others disagree (47). The evidence from animal studies is inconclusive regarding transmission of CJD between humans by transfusion, and study design and laboratory techniques are controversial.

### Evidence from Studies of Humans

### Human Case Series and Case Reports

Case series and case reports provided information linking CJD to receipt of dura mater and human growth hormone. However, no human cases have yet been causatively linked to blood transfusion. A transplant recipient developed CJD (48) after receiving a liver from a donor who died of a cerebrovascular accident and had no symptoms of CJD; however, an autopsy was not performed, and brain tissue was not available for further investigation. The liver recipient did not have any of the known PrP gene coding sequences pathognomonic of familial transmission. The donor also provided a heart (to a recipient who died shortly after surgery) and a kidney (to yet another recipient; it was removed after 2 months); the latter recipient remains well. The liver recipient also received transfusions of blood, plasma, albumin, and anticytomegalovirus immunoglobulin from other donors. None of the blood donors is known to have had CJD, but one of the albumin donors died of a rapidly developing undiagnosed dementia.

Four Australians have been reported with CJD following transfusion (49). The patients had cerebellar signs; however, no other evidence of iatrogenic cause was described (50). The source of blood transfusions was undocumented. Genetic testing results were not provided; it is uncertain if cases were of the familial type, and no other information on alternative iatrogenic sources was provided.

In Canada, an albumin recipient died of neuropathologically confirmed CJD after receiving albumin from a pool containing blood from a person who died of neuropathologically confirmed CJD (D.G. Patry, pers. comm.). Eight months separated the receipt of albumin and development of symptoms, a much shorter period by a factor of three than seen in any other putative iatrogenic case, which makes iatrogenic transmission unlikely. A complete investigation is under way.

Without an experimental, diagnostic, or epidemiologic tool that can distinguish sporadic from iatrogenic disease or link the agent from a source with a recipient, it is difficult to draw any conclusions from the case reports described above. Statistically, a certain number of persons exposed to pooled blood would be expected to develop CJD. In addition, bias is introduced into case reports because of strong suspicion regarding iatrogenic sources. Verifying the statistical probability of cases by calculating the expected number of cases is difficult, if not impossible, because CJD is rare.

### Surveillance

Population surveys or surveillance systems of the worldwide epidemiology of CJD indicate that CJD occurs in the population at a rate of 0.05 and 1.5 cases per million per year. After 1979, the rates are 0.3 to 1.5 cases per million per year (1-8). The age distribution patterns are consistent and show that cases in persons under 30 years of age are extremely rare; cases in persons under 50 years of age are rare; and the peak age of onset is 60 to 70 years. In age-specific data, rates decline in older age groups (2,6-8,51).

If CJD is transmissible in blood, cases should occur in young persons, particularly if the incubation period is short. Even if the incubation period is decades long, one would expect cases in young persons because of transfusions given to infants and children. However, CJD is rare in young persons and remains rare over time. Alternatively, the rare diagnosis of CJD in younger age groups may be due to preferences for neurologic disease diagnoses in these groups. Recent attention to nv-CJD in 14 persons under the age of 40 years in the United Kingdom, which has an intensive active surveillance system, will certainly affect investigations of unexplained mental deterioration among younger populations (22). The British Paediatric Surveillance System has initiated investigations into undiagnosed progressive neurologic disease among children (C. Verity, A. Nicoll and R. Will, pers. comm.), and Canada will initiate a similar system. In the United States, the Centers for Disease Control and Prevention has enhanced surveillance for CJD in persons under 55 years of age (52).

If CJD is transmitted in blood, the last three to four decades might show a detectable increase in cases reflecting the increasing use of blood transfusions. In fact, surveillance data demonstrate that CJD rates are increasing in some countries. Interpretation of this finding is difficult: most surveillance systems were initiated in the last two decades; in countries with intensive surveillance, “catch-up” from previous underreporting led to initial increases in case numbers and rates; few countries have sufficiently intensive surveillance systems to conclude that there is no risk from blood; and the death certificates used for surveillance in some countries are sometimes incomplete, which may introduce a bias toward easily ascertained cases (8,51).

If CJD is transmitted in pooled blood products, clusters would be detected; indeed, surveillance systems have expected such clusters. However, observation of one or two cases often leads to more careful search for cases. Most such clusters have been attributed to familial disease (1,53). Cluster investigations in surveillance systems have not systematically searched for blood-related transmission.

Surveillance systems have found cases of CJD among persons who have received blood transfusions, but none have been linked to blood transmission. In the United Kingdom, the well-established surveillance system identified nv-CJD, despite its rarity, demonstrating the capability of surveillance to find rare diseases; nv-CJD was recognized quickly because of the young age of the patients and the novel neuropathologic findings. However, the absence of evidence is not evidence of the absence of transmission of CJD through blood, for two reasons: 1) surveillance systems designed to identify rare diseases need intensive resource allocation to detect sentinel events and 2) blood-borne transmission may go unnoticed when examining population data if unaccompanied unique epidemiologic features (such as extreme youth or novel neuropathologic features).

If CJD is transmitted in blood, cases could increase in industrialized countries, where access to blood transfusion is greater. The number of reported cases is larger; however, cases have been found in every country in which they have been sought, although some countries have limited surveillance capacity. While some countries have higher rates of CJD, there is no evidence that this is due to transmissible forms of the disease. Rather, the higher rates are most likely due to older age distributions in industrialized countries' populations, surveillance biases following intensified surveillance for CJD, and familial clusters.

Finally, in many surveillance systems, the age distribution pattern for CJD is uniform (2,7,8,51). The pattern is more consistent with early exposure to an agent with long incubation periods, a common population exposure that peaked long ago and is now declining, or a heritable disorder that causes shortened life-span than with a risk which increases with age (e.g., spontaneous genetic mutation or stochastic change of the normal prion to the ß-pleated form). However, CJD may be uniformly underdiagnosed in older age groups; because of nv-CJD there will likely be increased attention to differential diagnoses among elderly persons dying of rapidly progressing dementing illnesses. We do not suggest that all sporadic cases are due to external exposure such as blood, but rather we draw attention to an important epidemiologic characteristic of CJD that is not consistent with an entirely stochastic or age-related event.

### Case Control Studies

In three countries, case-control studies of CJD have included questions regarding exposure to blood: Japan (54), the United Kingdom (55-58), and the United States (59). In the Japanese study, only one case-patient and three controls received blood transfusions. Two of the United Kingdom studies indicate no difference in exposure to blood between the case-patients and controls (55,58). A comparison of the frequency of receipt of blood among persons with CJD and controls matched for age and sex using data collected in the United Kingdom CJD surveillance system between 1980 and 1984 (56) showed no difference in the history of blood receipt between those with and without CJD. Although an odds ratio (OR) is not calculated for the data, our calculations indicate the OR is 0.78 (95% confidence interval 0.38, 1.58) with a power of only 30% to detect an OR different from one (60). Davinipour (mid-Atlantic United States) also found that blood exposure posed no risk, with an OR for blood transfusion of 0.6 (59). However, with only 26 cases, the confidence intervals or number of patients exposed was not mentioned, although the OR is described as significant. Wientjens et al. analyzed pooled data for 178 cases and 333 controls from three case-control studies (Japan, United States, United Kingdom) and did not find an OR different from one for blood transfusion (61).

Although not using a formal case-control study, Operskalski et al, compared rates of human immunodeficiency virus (HIV) dementia among three HIV-positive groups (persons with hemophilia, "other blood recipients," and "blood donors") using data from the Transfusion Safety Study (62). They hypothesized that if CJD was misdiagnosed as HIV dementia but was the true cause of dementia, there would be an excess rate of HIV dementia in persons with hemophilia due to their high rates of exposure to blood and blood products (persons with hemophilia were exposed to the blood or product made from pools of hundreds of thousands if not millions of donations). In addition, since the study contained data from as early as the 1950s, long periods of observation were available. Rates of HIV dementia in the study populations were compared, and CJD in pooled plasma derivatives did not pose a risk. However, it cannot be concluded that rare diseases such as CJD would be detectable in increased rates of misdiagnosed HIV dementia or that diseases with long incubation periods may have sufficient time to develop in persons with hemophilia. Any disease with an incubation period longer than that of HIV would be in competition with AIDS and hepatitis B or C as a cause of death, and persons with hemophilia and HIV infection die young. In addition, the data included the cause of death of 1,000 HIV-infected persons but did not provide information on duration of observation or an estimate of the rate of disease that would be necessary before even one additional case would be observed.

The primary weakness found in these published case-control studies is the use of the categories “exposure to blood” or “blood transfusion” as a surrogate for exposure to blood containing the agent of CJD. If the agent of CJD is frequently present in blood used for transfusions, the case-control study design is sufficiently robust to detect a risk. However, transmission of CJD through transfusion of blood contaminated with the agent of CJD appears to be rare. Consequently, a negative finding of risk attributable to blood in these studies may simply reflect an absence of exposure to the disease. Study design will have to account not only for a rare disease, but possibly for a rare exposure as well.

In addition, when hospitalized persons are used as controls, the design must consider Berkson’s bias, a selection bias that occurs when the selection method for controls affects the rate of exposure to the agent studied (63). The study design must then show that hospitalized controls do not have a different rate of potential exposure to blood transfusion from the general population. The use of population controls may be more appropriate for studying blood exposure.

Another weakness in the existing studies was the use of a reporter, most often a relative, to collect information about exposure to blood or blood products. Oral history of blood exposure tends to underestimate the rate of exposure to blood. In one Canadian hospital conducting a retrospective study, approximately 40% of patients did not know if they had received a blood transfusion (C. Kennealy, Pers. Comm.). In a central Ontario hospital, 25% of recipients did not know if they had received a blood transfusion (S. King, Pers. Comm.). Given that CJD and transfusion-transmitted CJD (if it occurs) are rare, the published case-control studies lack the power to detect very low ORs.

The 126 hemophilia centers in the United States have been asked to report deaths of persons with neurologic symptoms for neurologic confirmation of cause of death; no CJD cases have been reported in persons with hemophilia (B. Evatt and L. Schonberger, Pers. Comm.). The Canadian Haemophilia Society has requested assistance from the Health Canada, Laboratory Centre for Disease Control, to initiate a similar study in Canada (D. Wong-Reiger, Pers. Comm.). In Canada, a combined active surveillance system and case control study will begin in 1997 to identify the risk for CJD as a result of transfusion with blood from a person with CJD. Many of the methodologic problems in other studies have been addressed in the design of this study.

### Cohort Studies

The investigation of a patient with CJD who donated 35 units of blood in 20 years identified 27 persons who definitely received his blood and eight who probably received blood; for 20 units, the recipients could not be identified (64). Eighteen (33%) of the identified recipients had died. None of the recipients had exhibited neurologic disease, although some were observed only briefly; only eight were observed for longer than 5 years.

In the United States, the American Association of Blood Banks has initiated a long-term cohort investigation. By linking the names of persons who received blood from CJD patients with the national death records, the cause of death for each person will be determined. Of the 147 recipients identified, 80 have died; for 65, the cause of death is known but no cases of CJD have been found (M. Sullivan, Pers. Comm.).

Cohort studies are unlikely to accumulate enough cases of exposure to allow a reasonably precise estimate of risk. However, if pooled product confers uniform risk to each recipient, data from a large number of exposed recipients can be collected. The cohort studies under way may be able to identify higher than expected rates of disease, thus indicating transmission through blood transfusion without quantifying the rate of transmission.

### Conclusions

Many health professionals are concerned that CJD may be transmissible through blood. As noted by Brown, "iatrogenic disease from this source would dwarf in importance all other sources by virtue of the sheer number of people who theoretically have been or could be at risk" (27).

Animal studies indicate that the infective agent of CJD is present in blood but in low titer, and sufficient evidence of animal transmission suggests that the disease has the potential to be transmitted through blood (65). However, human epidemiologic evidence only indicates that if blood transmission occurs, it is likely rare. Some researchers suspect that the agent of CJD is ubiquitous, therefore, we are commonly exposed to it; perhaps, another, as yet unidentified factor, "turns on" the disease. If so, is there any part of the human population that is not exposed? Most people are exposed to some components of blood products through vaccines. Some people may be protected by strain variation through exposure to nonvirulent forms, so only subgroups are susceptible to a virulent form (66). Polymorphism at codon 129 may restrict susceptibility to a small portion of the population, or most transfusions may not contain a dose large enough to cause infection.

Can experimental studies answer these questions? Sufficiently large numbers of study animals could overcome problems such as the species barrier and low transmission rates due to a low dose or peripheral route of inoculation. The studies would require strict adherence to proper laboratory technique and replication in at least one other laboratory. Unanswered questions include the following: Can the transmissible spongiform encephalopathies of animals be transmitted, by transfusion, within the species when spiked blood is used? Within species from "naturally infected" blood? From humans to animals with spiked blood? From humans to animals with "naturally infected" blood? Does transmission by blood become more efficient after passage? Can human blood cells carry the agent? What are the routes for infection of the brain if infection is peripheral?

Can epidemiologic methods detect blood transmission of CJD if it is rare? Epidemiologic tools such as outbreak investigations, case-control studies, and cohort studies are limited in their ability to detect rare events. In addition, during the long incubation period, patients often move away from the location where they received the transfusion; forget their exposure; and essentially lose their membership in a recognizable cohort. The absence of a test for exposure, such as an antibody test or gene sequencing of an agent as used in investigating HIV-related outbreaks, makes the investigation of iatrogenic CJD extremely difficult, hence the importance of vigilance, in the form of case reports from alert and informed clinicians, followed by critical field investigation. Surveillance systems provide the core resources for identifying and investigating unique or unexplained events. Followup case-control studies allow the observation and recording of the actual chain of exposure to blood.

### Acknowledgments

The authors thank Drs. Paul Gully, Jamie Hockin, and Martin Tepper for their thoughtful reviews of earlier drafts of this paper and Drs. Catherine Bergeron, Michael Coulthart, and Brian Foster for discussion and scientific assistance.

Dr. Ricketts is a medical specialist in the Blood-borne Pathogens Division, Laboratory Centre for Diseases Control, Ottawa, Ontario, Canada. Her research interests include the CJD surveillance system in Canada, a collaborative international study on the incidence and risk factors for CJD, and policy and infection control guidelines for CJD and other prion diseases.

### References

1. Masters CL, Harris JO, Gajdusek C, Gibbs CJ, Bernoulli C, Asher DM. Creutzfeldt-Jakob disease: patterns of worldwide occurrence and the significance of familial and sporadic clustering. Ann Neurol 1979;5:177-88.
2. Brown P, Cathala F, Raubertas RF, Gajdusek DC, Castaigne P. The epidemiology of Creutzfeldt-Jakob disease: conclusion of a 15 year investigation in France and review of the world literature. Neurology 1987;37:895-904.
3. Will RG. Incidence of Creutzfeldt-Jakob disease in the European Community. In: Gibbs CJ Jr, editor. Bovine Spongiform Encephalopathy: The BSE Dilemma. New York: Springer-Verlag 1996:364-74.
4. World Health Organization consultation on clinical and neuropathological characteristics of the new variant of CJD and other human and animal TSEs. Geneva: WHO, 1996.

5. Creutzfeldt-Jakob disease in Australia: first annual report. Melbourne: The National CJD Registry, 1996.
6. Stratton E, Ricketts MN, Gully PR. Creutzfeldt-Jakob disease in Canada. CCDR 1996;22:57-61.
7. Kovanen J, Haltia M. Descriptive epidemiology of Creutzfeldt-Jakob disease in Finland. Acta Neurol Scand 1980;77:474-80.
8. Holman RC, Khan AS, Kent J, Strine TW, Schonberger LB. Epidemiology of Creutzfeldt-Jakob disease in the United States, 1979-1990: analysis of national mortality data. Neuroepidemiology 1995;14:174-81.
9. de Silva R. Human spongiform encephalopathy: clinical presentation and diagnostic tests. In: Baker H, Ridley RM, editors. Methods in molecular medicine: prion diseases. Totawa (NJ): Humana Press Inc; 1996:15-33.
10. Kretzschmar HA, Ironside JW, DeArmond SJ, Tateishi J. Diagnostic criteria for sporadic Creutzfeldt-Jakob disease. Arch Neurol 1996;53:913-20.
11. Ironside JW. Neuropathological diagnosis of human prion disease. In: Baker H, Ridley RM, editors. Methods in molecular medicine: prion diseases. Totawa (NJ): Humana Press Inc; 1996:35-57.
12. Parchi P, Castellani R, Capellari S, Ghetti B, Young K, Chen SG, et al. Molecular basis of phenotypic variability in sporadic Creutzfeldt-Jakob disease. Ann Neurol 1996;39:767-78.
13. Collinge J, Sidle DC, Meads J, Ironside J, Hill AF. Molecular analysis of prion strain variation and the aetiology of "new variant" CJD. Nature 1996;383:685-90.
14. Zerr I, Bodemer M, Otto M, Poser S, Wind IO, Kretzschmar HA, Poser S, Wind IO, Kretzschmar HA, et al. Diagnosis of Creutzfeldt-Jakob disease by two-dimensional gel electrophoresis of cerebrospinal fluid. Lancet 1996;348:846-9.
15. Hsich G, Kenney K, Gibbs CJ, Lee KH, Harrington MG. The 14-2-2 brain protein in cerebrospinal fluid as a marker for transmissible spongiform encephalopathies. N Engl J Med 1996;335:924-30.
16. Manuelidis L. The dimensions of Creutzfeldt-Jakob disease. Transfusion 1994;34:915-28.
17. Ozel M, Diringer H. Small virus-like structure in fractions from scrapie hamster brain. Lancet 1994;343:894-5.
18. Brown P, Preece MA, Will, RG. "Friendly Fire" in medicine: hormones, homografts and CJD. Lancet 1992;340:24-7.
19. Lasmézas CI, Deslys JP, Robain O, et al. Transmission of the BSE agent to mice in the absence of detectable abnormal prion protein. Science 1997;275:402-5.
20. Collinge J, Palmer MS, Dryden AJ. Genetic predisposition to iatrogenic Creutzfeldt-Jakob disease. Lancet 1991;337:1441-2.
21. Brown P, Gajdusek DC. The Human Spongiform Encephalopathies: Kuru, Creutzfeldt-Jakob Disease, and the Gerstmann-Straussler-Scheinker Syndrome. In: Chesebro BW, editor. Current topics in microbiology and immunology, vol 172. New York: Springer-Verlag, 1991:1-20.
22. Will RG, Ironside JW, Zeidler M, Cousens SN, Estibeiro K, Alperovitch A, et al. A new variant of Creutzfeldt-Jakob disease in the UK. Lancet 1996;347:921-5.
23. Gajdusek DC, Gibbs CJ, Alpers M. Experimental transmission of a kuru-like syndrome to chimpanzees. Nature 1966;209:794-6.
24. Gibbs CJ Jr, Gajdusek DC, Asher DM, Alpers MP, Beck E, Daniel PM, Matthews WB. Creutzfeldt-Jakob disease (subacute spongiform encephalopathy): transmission to the chimpanzee. Science 1968;161:388-9.
25. Duffy P, Wolf J, Collins G, DeVoe AG, Streeten B, Cowen D. Possible person-person transmission of CJD. N Engl J Med 1974;290:692.
26. Liberski PP, Yanagihara R, Gibbs CJ, Gajdusek DC. Spread of Creutzfeldt-Jakob disease virus along visual pathways after intra ocular inoculation. Arch Virol 1990;111:141-7.
27. Maneulidis EE, Maneulidis L. Experiments on maternal transmission of Creutzfeldt-Jakob disease in guinea pigs. Proc Soc Exp Biol Med 1979;160:233-6.
28. Brown P. Environmental causes of human spongiform encephalopathy. In: Baker H, Ridley RM, editors. Methods in molecular medicine: prion diseases. Totawa (NJ): Humana Press Inc; 1996:139-54.
29. Bernoulli C, Siegfried J, Baumgartner G, Regli F, Rabinowicz T, Gajdusek DC, et al. Danger of accidental person-to-person transmission of CJD by surgery. Lancet 1977;1:478-9.
30. Brown P. Transmissible human spongiform encephalopathy (infectious cerebral amyloidosis): Creutzfeldt-Jakob disease, Gerstmann-Straussler-Scheinker's syndrome and kuru. In: Calne DB, editor. Neurodegenerative diseases. Philadelphia: 1994:839-76.
31. Foncin J, Gaches J, Cathala F, El Sharif E, Le Beau J. Transmission iatrogene interhumaine possible de maladie de Creutzfeldt-Jakob disease avec atteinte des grains du cervelet. Rev Neurol 1980;136:280.
32. Will RG, Matthews WB. Evidence for case-to-case transmission of Creutzfeldt-Jakob disease. J Neurol Neurosurg Psychiat 1982;45:235-8.
33. Nevin S, McMenemy WH, Berhman D, Jones DP. Subacute spongiform encephalopathy: a subacute form of encephalopathy attributable to vascular dysfunction (spongiform cerebral atrophy). Brain 1960;83:519-64.
34. Prichard J, Thadani V, Kalb R, Manuelidis E, Holder J. Rapidly progressive dementia in a patient who received a cadaveric dura mater graft. MMWR Morb Moral Wkly Rep 1987;36:49-50, 55.
35. Centers for Disease Control. Update: Creutzfeldt-Jakob disease in a second patient who received a cadaveric dura mater graft. MMWR Morb Moral Wkly Rep 1989;38:37-8, 43.
36. Esmonde T, Lueck CJD, Symon L, Duchen LW, Will RG. Creutzfeldt-Jakob disease and lyophilised dura mater grafts: report of two cases. J Neurol Neurosurg Psychiat. 1994;56:999-1000.
37. Centers for Disease Control. Fatal degenerative neurologic disease in patients who received pituitary derived human growth hormone. MMWR Morb Moral Wkly Rep 1985;34:359-60, 365-6.
38. Cochius JJ, Hyman N, Esiri MM. Creutzfeldt-Jakob disease in a recipient of human pituitary-derived gonadotropin, a second case. J Neurol Neurosurg Psych 1992;55:1094-5.

39. Brown P, Cervenakova L, Goldfarb LG, McCombie WR, Rubenstein R, Will RG, et al. Iatrogenic Creutzfeldt-Jakob disease: an example of the interplay between ancient genes and modern medicine. Neurology 1994;44:291-3.
40. Manuelidis EE, Kim JH, Mericangas JR, Manuelidis L. Transmission to animals of Creutzfeldt-Jakob disease from human blood. Lancet 1985;2:896-7.
41. Tateishi J. Transmission of Creutzfeldt-Jakob disease from human blood and urine into mice. Lancet 1985;2:1074.
42. Brown P, Gibbs CJ, Rodgers-Johnson P, Asher DM, Sulima MP, Bacote A, et al. Human spongiform encephalopathy: the NIH series of 300 cases of experimentally transmitted disease. Ann Neurol 1994;44:513-5.
43. Lavelle GC, Sturman L, Hadlow WJ. Isolation from mouse spleen of cell populations with high specific infectivity for scrapie virus. Infect Immun 1972;5:319-23.
44. Kuroda Y, Gibbs CJ, Amyx HL, Gajdusek DC. Creutzfeldt-Jakob disease in mice: persistent viraemia and preferential replication of virus in low density lymphocytes. Infect Immun 1983;41:154-61.
45. Czub M, Braig HR, Blode H, Diringer H. The major protein of SAF is absent from spleen and thus not an essential part of the scrapie agent. Arch Virol 1986;91:83-6.
46. Manuelidis EE, Gorgacs EJ, Manuelidis L. Viraemia in experimental Creutzfeldt-Jakob disease. Science 1978;200:1069-71.
47. Deslys JP, Lasmezas C, Dormont D. Selection of specific strains in iatrogenic Creutzfeldt-Jakob disease (letter) Lancet 1994;343:848-9.
48. Matthews WB. Transmission of Creutzfeldt-Jakob disease (letter). Lancet 1994;343:1575-6.
49. Creange A, Gray F, Cesaro, Adle-Biassette H, Duvoux C, Cherqui D, et al. Creutzfeldt-Jakob disease after liver transplantation. Ann Neurol 1995;38:269-72.
50. Klein R, Dumble LJ. Transmission of Creutzfeldt-Jakob disease by blood transfusion. Lancet. 1993;341:768.
51. Collins S, Masters CL. Iatrogenic and zoonotic Creutzfeldt-Jakob disease: the Australian perspective. Med J Aust 1996;164:598-602.
52. Will RG. Surveillance of prion diseases in humans. In: Baker H, Ridley RM, editors. Methods in molecular medicine: prion diseases. Totawa (NJ): Humana Press Inc., 1996;119-37.
53. Reingold A, Rothrock G, Starr M, Reilly K, Vugia D, Waterman S, et al. Surveillance for Creutzfeldt-Jakob disease-United States. MMWR Morb Moral Wkly Rep 1996;45:665-8.
54. Raubertas RF, Brown P, Cathala F, Brown I. The Question of clustering of Creutzfeldt-Jakob disease. Am J Epidemiol 1989;129:146-54.
55. Kondo K, Kuroiwa Y. A case control study of Creutzfeldt-Jakob disease: association with physical injuries. Ann Neurol 1982;11:377-81.
56. Will RG. Epidemiological surveillance of Creutzfeldt-Jakob disease in the United Kingdom. Eur J Epidemiol 1991;7:460-5.
57. Esmonde TFG, Will RG, Slattery JM, Knight R, Harries-Jones R, de Silva R, et al. Creutzfeldt-Jakob disease and blood transfusion. Lancet 1993;341:205-7.
58. Harries-Jones R, Knight R, Will RG, Cousens S, Smith PG, Matthews WB. Creutzfeldt-Jakob disease in England and Wales, 1980-1984: a case-control study of potential risk factors. J Neurol Neurosurg Psychiatry 1988;51:1113-9.
59. Esmonde TFG, Ireland BN, Will RG, Ironside J. Creutzfeldt-Jakob disease: A case-control study. Neurology 1994;44:A193 (Abstract 260P)
60. Davanipour Z, Alter M, Sobel E, Asher D, Gajdusek DC. Creutzfeldt-Jakob disease: possible medical risk factors. Neurology 1985;35:1483-6.
61. Walter SD. Determination of significant relative risks and optimal sampling procedures in prospective and retrospective comparative studies of various sizes. Am J Epidemiol. 1977;105:387-97.
62. Wientjens DPWM, Davinipour Z, Hofman A, Kondo K, Matthews WB, Will RG, van Duijn CM. Risk factors for Creutzfeldt-Jakob disease: a reanalysis of case-control studies. Neurology 1996;46:1287-91.
63. Operskalski EA, Mosley JW. Pooled plasma derivatives and Creutzfeldt-Jakob disease. Lancet 1995;346:1223.
64. Schlesselman JJ, Stolley PD. Sources of bias. In: Schlesselman JJ, editor. Case-control studies: design, conduct, analysis. New York: Oxford University Press, 1982:124-43.
65. Heye N, Hensen S, Muller N. Creutzfeldt-Jakob disease and blood transfusion. Lancet 1994;343:298-9.
66. Brown P. Can Creutzfeldt-Jakob disease be transmitted by transfusion? Current Opinion in Hematology 1995;2:472-7.
67. Deslys JP, Lasmezas CI, Billette de Villemeur T, Jaegly A, Dormont D. Creutzfeldt-Jakob disease. Lancet 1996;347:1332.

## References

[PDF_00805] Bernoulli C., Siegfried J., Baumgartner G., Regli F., Rabinowicz T., Gajdusek D. C., Gibbs C. J. (1977). Danger of accidental person-to-person transmission of Creutzfeldt-Jakob disease by surgery.. Lancet.

[PDF_00707] Brown P., Cathala F., Raubertas R. F., Gajdusek D. C., Castaigne P. (1987). The epidemiology of Creutzfeldt-Jakob disease: conclusion of a 15-year investigation in France and review of the world literature.. Neurology.

[PDF_00847] Brown P., Cervenáková L., Goldfarb L. G., McCombie W. R., Rubenstein R., Will R. G., Pocchiari M., Martinez-Lage J. F., Scalici C., Masullo C. (1994). Iatrogenic Creutzfeldt-Jakob disease: an example of the interplay between ancient genes and modern medicine.. Neurology.

[PDF_00858] Brown P., Gibbs C. J., Rodgers-Johnson P., Asher D. M., Sulima M. P., Bacote A., Goldfarb L. G., Gajdusek D. C. (1994). Human spongiform encephalopathy: the National Institutes of Health series of 300 cases of experimentally transmitted disease.. Ann Neurol.

[PDF_00766] Brown P., Preece M. A., Will R. G. (1992). "Friendly fire" in medicine: hormones, homografts, and Creutzfeldt-Jakob disease.. Lancet.

[PDF_00837] Centers for Disease Control (CDC) (1985). Fatal degenerative neurologic disease in patients who received pituitary-derived human growth hormone.. MMWR Morb Mortal Wkly Rep.

[PDF_00829] Centers for Disease Control (CDC) (1989). Update: Creutzfeldt-Jakob disease in a second patient who received a cadaveric dura mater graft.. MMWR Morb Mortal Wkly Rep.

[PDF_00841] Cochius J. I., Hyman N., Esiri M. M. (1992). Creutzfeldt-Jakob disease in a recipient of human pituitary-derived gonadotrophin: a second case.. J Neurol Neurosurg Psychiatry.

[PDF_00772] Collinge J., Palmer M. S., Dryden A. J. (1991). Genetic predisposition to iatrogenic Creutzfeldt-Jakob disease.. Lancet.

[PDF_00749] Collinge J., Sidle K. C., Meads J., Ironside J., Hill A. F. (1996). Molecular analysis of prion strain variation and the aetiology of 'new variant' CJD.. Nature.

[PDF_00888] Collins S., Masters C. L. (1996). Iatrogenic and zoonotic Creutzfeldt-Jakob disease: the Australian perspective.. Med J Aust.

[PDF_00882] Créange A., Gray F., Cesaro P., Adle-Biassette H., Duvoux C., Cherqui D., Bell J., Parchi P., Gambetti P., Degos J. D. (1995). Creutzfeldt-Jakob disease after liver transplantation.. Ann Neurol.

[PDF_00919] Davanipour Z., Alter M., Sobel E., Asher D., Gajdusek D. C. (1985). Creutzfeldt-Jakob disease: possible medical risk factors.. Neurology.

[PDF_00941] Deslys J. P., Lasmezas C. I., Billette de Villemeur T., Jaegly A., Dormont D. (1996). Creutzfeldt-Jakob disease.. Lancet.

[PDF_00877] Deslys J. P., Lasmézas C., Dormont D. (1994). Selection of specific strains in iatrogenic Creutzfeldt-Jakob disease.. Lancet.

[PDF_00791] Duffy P., Wolf J., Collins G., DeVoe A. G., Streeten B., Cowen D. (1974). Letter: Possible person-to-person transmission of Creutzfeldt-Jakob disease.. N Engl J Med.

[PDF_00908] Esmonde T. F., Will R. G., Slattery J. M., Knight R., Harries-Jones R., de Silva R., Matthews W. B. (1993). Creutzfeldt-Jakob disease and blood transfusion.. Lancet.

[PDF_00833] Esmonde T., Lueck C. J., Symon L., Duchen L. W., Will R. G. (1993). Creutzfeldt-Jakob disease and lyophilised dura mater grafts: report of two cases.. J Neurol Neurosurg Psychiatry.

[PDF_00784] Gajdusek D. C., Gibbs C. J., Alpers M. (1966). Experimental transmission of a Kuru-like syndrome to chimpanzees.. Nature.

[PDF_00787] Gibbs C. J., Gajdusek D. C., Asher D. M., Alpers M. P., Beck E., Daniel P. M., Matthews W. B. (1968). Creutzfeldt-Jakob disease (spongiform encephalopathy): transmission to the chimpanzee.. Science.

[PDF_00911] Harries-Jones R., Knight R., Will R. G., Cousens S., Smith P. G., Matthews W. B. (1988). Creutzfeldt-Jakob disease in England and Wales, 1980-1984: a case-control study of potential risk factors.. J Neurol Neurosurg Psychiatry.

[PDF_00936] Heye N., Hensen S., Müller N. (1994). Creutzfeldt-Jakob disease and blood transfusion.. Lancet.

[PDF_00729] Holman R. C., Khan A. S., Kent J., Strine T. W., Schonberger L. B. (1995). Epidemiology of Creutzfeldt-Jakob disease in the United States, 1979-1990: analysis of national mortality data.. Neuroepidemiology.

[PDF_00757] Hsich G., Kenney K., Gibbs C. J., Lee K. H., Harrington M. G. (1996). The 14-3-3 brain protein in cerebrospinal fluid as a marker for transmissible spongiform encephalopathies.. N Engl J Med.

[PDF_00885] Klein R., Dumble L. J. (1993). Transmission of Creutzfeldt-Jakob disease by blood transfusion.. Lancet.

[PDF_00902] Kondo K., Kuroiwa Y. (1982). A case control study of Creutzfeldt-Jakob disease: association with physical injuries.. Ann Neurol.

[PDF_00726] Kovanen J., Haltia M. (1988). Descriptive epidemiology of Creutzfeldt-Jakob disease in Finland.. Acta Neurol Scand.

[PDF_00738] Kretzschmar H. A., Ironside J. W., DeArmond S. J., Tateishi J. (1996). Diagnostic criteria for sporadic Creutzfeldt-Jakob disease.. Arch Neurol.

[PDF_00866] Kuroda Y., Gibbs C. J., Amyx H. L., Gajdusek D. C. (1983). Creutzfeldt-Jakob disease in mice: persistent viremia and preferential replication of virus in low-density lymphocytes.. Infect Immun.

[PDF_00769] Lasmézas C. I., Deslys J. P., Robain O., Jaegly A., Beringue V., Peyrin J. M., Fournier J. G., Hauw J. J., Rossier J., Dormont D. (1997). Transmission of the BSE agent to mice in the absence of detectable abnormal prion protein.. Science.

[PDF_00863] Lavelle G. C., Sturman L., Hadlow W. J. (1972). Isolation from mouse spleen of cell populations with high specific infectivity for scrapie virus.. Infect Immun.

[PDF_00794] Liberski P. P., Yanagihara R., Gibbs C. J., Gajdusek D. C. (1990). Spread of Creutzfeldt-Jakob disease virus along visual pathways after intraocular inoculation.. Arch Virol.

[PDF_00874] Manuelidis E. E., Gorgacs E. J., Manuelidis L. (1978). Viremia in experimental Creutzfeldt-Jakob disease.. Science.

[PDF_00852] Manuelidis E. E., Kim J. H., Mericangas J. R., Manuelidis L. (1985). Transmission to animals of Creutzfeldt-Jakob disease from human blood.. Lancet.

[PDF_00761] Manuelidis L. (1994). The dimensions of Creutzfeldt-Jakob disease.. Transfusion.

[PDF_00702] Masters C. L., Harris J. O., Gajdusek D. C., Gibbs C. J., Bernoulli C., Asher D. M. (1979). Creutzfeldt-Jakob disease: patterns of worldwide occurrence and the significance of familial and sporadic clustering.. Ann Neurol.

[PDF_00880] Matthews W. B. (1994). Transmission of Creutzfeld-Jakob disease.. Lancet.

[PDF_00821] NEVIN S., McMENEMEY W. H., BEHRMAN S., JONES D. P. (1960). Subacute spongiform encephalopathy--a subacute form of encephalopathy attributable to vascular dysfunction (spongiform cerebral atrophy).. Brain.

[PDF_00763] Ozel M., Diringer H. (1994). Small virus-like structure in fractions from scrapie hamster brain.. Lancet.

[PDF_00745] Parchi P., Castellani R., Capellari S., Ghetti B., Young K., Chen S. G., Farlow M., Dickson D. W., Sima A. A., Trojanowski J. Q. (1996). Molecular basis of phenotypic variability in sporadic Creutzfeldt-Jakob disease.. Ann Neurol.

[PDF_00899] Raubertas R. F., Brown P., Cathala F., Brown I. (1989). The question of clustering of Creutzfeldt-Jakob disease.. Am J Epidemiol.

[PDF_00855] Tateishi J. (1985). Transmission of Creutzfeldt-Jakob disease from human blood and urine into mice.. Lancet.

[PDF_00922] Walter S. D. (1977). Determination of significant relative risks and optimal sampling procedures in prospective and retrospective comparative studies of various sizes.. Am J Epidemiol.

[PDF_00926] Wientjens D. P., Davanipour Z., Hofman A., Kondo K., Matthews W. B., Will R. G., van Duijn C. M. (1996). Risk factors for Creutzfeldt-Jakob disease: a reanalysis of case-control studies.. Neurology.

[PDF_00781] Will R. G., Ironside J. W., Zeidler M., Cousens S. N., Estibeiro K., Alperovitch A., Poser S., Pocchiari M., Hofman A., Smith P. G. (1996). A new variant of Creutzfeldt-Jakob disease in the UK.. Lancet.

[PDF_00818] Will R. G., Matthews W. B. (1982). Evidence for case-to-case transmission of Creutzfeldt-Jakob disease.. J Neurol Neurosurg Psychiatry.

[PDF_00752] Zerr I., Bodemer M., Otto M., Poser S., Windl O., Kretzschmar H. A., Gefeller O., Weber T. (1996). Diagnosis of Creutzfeldt-Jakob disease by two-dimensional gel electrophoresis of cerebrospinal fluid.. Lancet.

